# Nonlinear association of a composite metabolic index (ZJU index) with hypertension: a cross-sectional study of NHANES 2003–2018

**DOI:** 10.3389/fcvm.2025.1608648

**Published:** 2025-06-18

**Authors:** Haibo Gong, Jing Chen, Xiao Chen, Yuanhe Fan, Yuan Luo

**Affiliations:** ^1^ Department of Rehabilitation, The First People’s Hospital of Neijiang, Neijiang, Sichuan, China; ^2^ Department of Pediatrics, The First People’s Hospital of Neijiang, Neijiang, Sichuan, China; ^3^ Department of Orthopedics, The First People’s Hospital of Neijiang, Neijiang, Sichuan, China

**Keywords:** ZJU index, hypertension, NHANES, risk, cross-sectional study

## Abstract

**Objective:**

To explore the association between the composite metabolic index (ZJU index) and hypertension using data from the National Health and Nutrition Examination Survey (NHANES).

**Methods:**

NHANES data from 2003 to 2018 were analyzed. Participants were categorized into hypertension and non-hypertension groups. Logistic regression models evaluated the relationship between ZJU index and hypertension. Restricted cubic spline (RCS) and threshold effect analyses assessed nonlinear associations. Subgroup and interaction analyses tested robustness and heterogeneity. The predictive ability of the ZJU index across age groups was evaluated using receiver operating characteristic (ROC) curves.

**Results:**

After adjusting for covariates, each unit increase in ZJU index was associated with a 7% higher odds of hypertension (OR = 1.07; 95% CI: 1.06–1.07). Participants with higher ZJU index values had significantly increased risk compared to the reference group (OR = 3.73; 95% CI: 3.25–4.29). RCS analyses indicated a nonlinear positive association, with a threshold inflection point at 53.22. Subgroup analyses confirmed consistent associations across all subgroups, while significant interactions were observed for age, education, diabetes history, and smoking status (*P* < 0.05). The ZJU index showed moderate predictive ability in individuals under 60 years (AUC = 0.691) and low predictive value in those aged 60 and above (AUC = 0.604).

**Conclusions:**

An elevated ZJU index is significantly associated with increased hypertension risk among U.S. adults, with a nonlinear dose-response relationship observed.

## 1 Introduction

The disease burden of hypertension, a major public health problem worldwide, has risen significantly over the past few decades (1). Despite advances in diagnostic and therapeutic techniques and medications, many people still fail to have their blood pressure diagnosed in a timely manner or effectively controlled (2, 3). This situation not only increases the health burden of individual patients, but also has a broad impact on cardiovascular disease (CVD) morbidity and mortality (4–7). Hypertension is one of the most important controllable risk factors for complications such as ischemic heart disease, stroke, and renal failure, and poses a serious threat to public health (8–11). Although existing strategies have made some progress in improving early detection, relevant risk assessment tools still face many challenges, especially in the development of non-invasive and cost-effective assessment methods (12–15). Therefore, there is an urgent need to find novel metabolism-related markers to optimize early risk identification and stratification strategies for hypertension.

Among the various metabolic indices developed to assess cardiometabolic risk, the homeostasis model assessment of insulin resistance (HOMA-IR) and the triglyceride-glucose (TyG) index have been widely utilized (16–19). However, these markers typically focus on a single metabolic pathway such as insulin resistance or dysglycemia, and may not fully reflect the complex, multidimensional nature of metabolic health related to hypertension. In recent years, the ZJU index has received increasing attention as an emerging comprehensive metabolic marker. The index is able to reflect the metabolic health level of an individual in a multidimensional way by integrating several metabolism-related indexes, including body mass index (BMI), fasting blood glucose (FBG), triglyceride (TG), alanine aminotransferase/aspartate aminotransferase ratio (ALT/AST), and a gender correction factor (20, 21). Unlike HOMA-IR or TyG, the ZJU index simultaneously incorporates factors of obesity, glucose and lipid metabolism, liver function, and sex differences, thus providing a more comprehensive depiction of an individual's metabolic status. Each of these components is closely associated with established mechanisms underlying hypertension, such as insulin resistance, systemic inflammation, obesity, and hepatic dysfunction (22, 23). For example, the ALT/AST ratio serves as a surrogate for liver injury, and abnormalities in liver function are often accompanied by hyperactivation of inflammatory responses and endothelial dysfunction, all of which have a significant impact on blood pressure regulation (24–26). The ZJU index has previously been shown to demonstrate superior efficacy over traditional metabolic indices (such as the visceral adiposity index and the fatty liver index) in the assessment of risk for nonalcoholic fatty liver disease (NAFLD) (27). The index has also been shown to be effective in the assessment of risk for type 2 diabetes mellitus. It has also shown potential value in the prediction of type 2 diabetes and cardiovascular comorbidities (28). However, existing studies have paid less attention to the specific relationship between the ZJU index and hypertension, especially its nonlinear effect in blood pressure and its applicability as a risk stratification tool, which still needs further validation.

Nevertheless, most existing research has either focused on indices such as HOMA-IR and TyG or studied the ZJU index in the context of liver disease and diabetes, while the direct association between the ZJU index and hypertension remains largely unexplored. To our knowledge, no previous study has systematically examined the potential nonlinear relationship between the ZJU index and hypertension, or evaluated its performance as a risk stratification tool in diverse populations. Therefore, the present study, utilizing a representative U.S. adult population from the National Health and Nutrition Examination Survey (NHANES), aims to fill this research gap by investigating the association between the ZJU index and hypertension, as well as its predictive value for hypertension risk. This work is expected to provide important evidence to support the development of more accurate early risk assessment and management strategies for hypertension.

## 2 Methods

### 2.1 Study design and population

The present study utilized data from the ongoing NHANES, which is conducted by the National Center for Health Statistics, a division of the Centers for Disease Control and Prevention (CDC). For more detailed information regarding NHANES, please refer to the CDC's dedicated website (http://www.cdc.gov/nchs/nhanes/). NHANES employs multi-stage probability sampling methodologies to gather comprehensive data from a representative sample of the non-institutionalized U.S. civilian population. Ethical approval for the original NHANES data collection was granted by the Institutional Review Board of the National Center for Health Statistics (NCHS), with informed consent obtained from all participants. In accordance with ethical guidelines, de-identified NHANES data are publicly accessible. In this study, researchers who were not involved in the initial NHANES program accessed and analyzed the data; thus, secondary analysis did not necessitate additional ethical approval.

**Figure 1:**
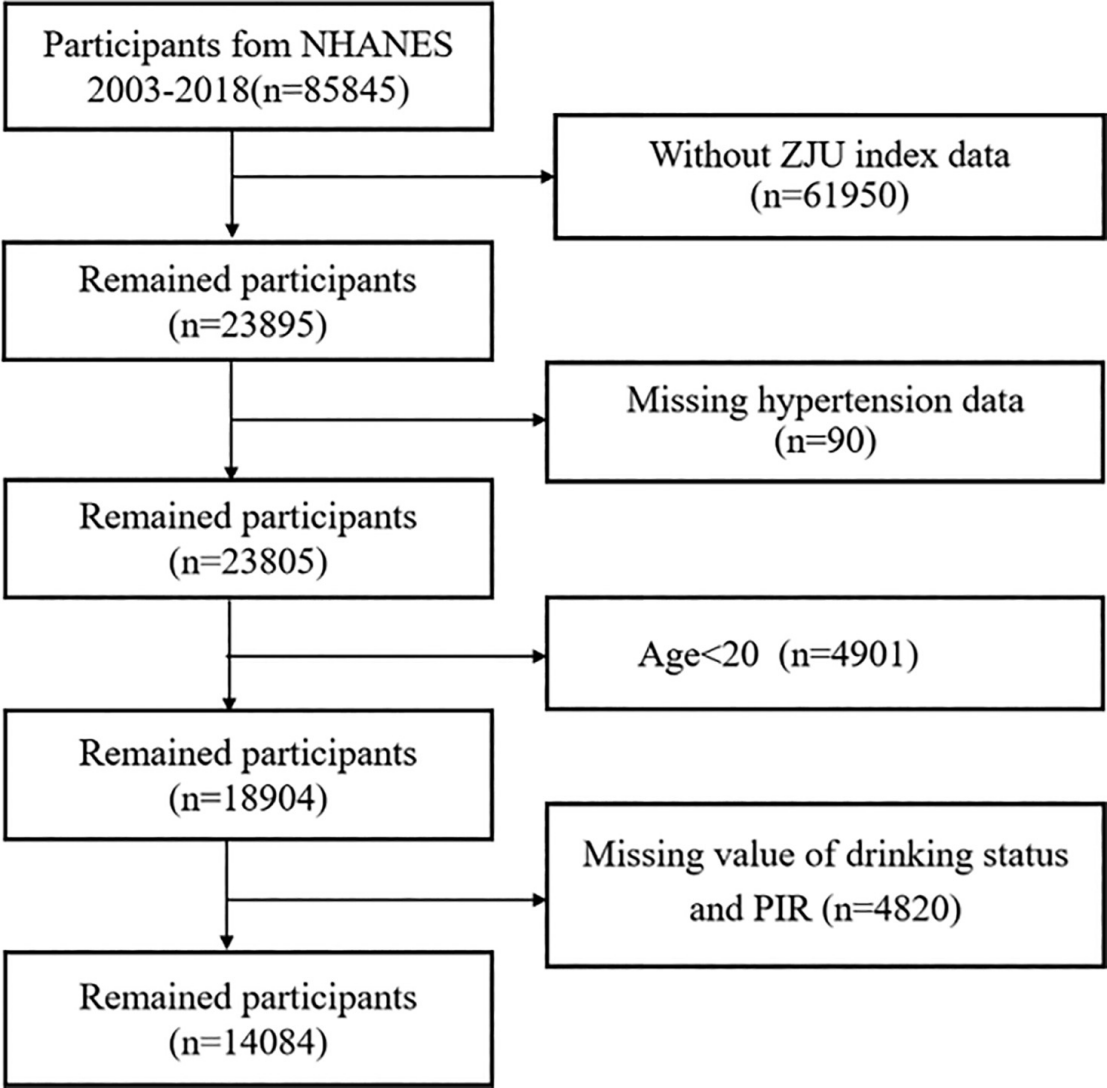
The flowchart of study participants.

Data from eight survey cycles spanning 2003 to 2018 were selected to investigate the relationship between the ZJU index and the prevalence of hypertension. Figure 1 illustrates the participant selection process. Initially, individuals without ZJU index records (*n* = 61950) and those with incomplete hypertension data (*n* = 90) were excluded. Additionally, individuals under the age of 20 (*n* = 4901), those with missing dringking status and PIR data (*n* = 4820) were excluded from the analysis. Participants with missing data on PIR and drinking status were excluded because these variables are important socioeconomic and lifestyle factors, respectively. Incomplete data on these could lead to residual confounding and reduced model validity, as both factors are known to influence hypertension risk and were included as essential covariates in the regression models. The resulting dataset comprised 14,084 individuals with complete information, of whom 5,976 had hypertension.

### 2.2 Definition of ZJU index

The ZJU index was calculated as originally proposed by [Wang et al. (21)]:

ZJU index = FPG (mmol/L) + BMI(kg/m^2^) + 3*ALT(U/L)/AST(U/L)ratio (+2 if female) + TG (mmol/L) (21, 28, 29).

### 2.3 Definition of hypertension

Blood pressure measurements were taken by trained health professionals using a mercury sphygmomanometer in the Mobile Examination Centre (MEC). Measurements were taken in a sitting position, mainly using the right arm, unless otherwise stated. After participants rested for 5 min, 3 consecutive blood pressure readings were taken and the average of these 3 readings was used for subsequent analysis. Hypertension was defined as systolic blood pressure ≥140 mmHg, diastolic blood pressure ≥90 mmHg, or self-reported physician-diagnosed hypertension or use of antihypertensive medication (30).

### 2.4 Covariables

To minimise potential confounding when analysing the relationship between the ZJU index and hypertension, we used a multivariate adjustment model. Covariates included in the multivariate models were selected based on previous literature and known associations with both metabolic indices and hypertension, aiming to adjust for potential confounding. Demographic (age, gender, race, education level, PIR), health status (diabetes, cardiovascular disease, cancer), laboratory measures (LDL, HDL, TC), and lifestyle factors (smoking, alcohol consumption) were all included, as each has been independently associated with hypertension risk in prior large-scale epidemiological studies.PIR is calculated by dividing household or individual income by the poverty threshold adjusted annually for inflation and family size, serving as a measure of socioeconomic status. Participants are categorized into three groups based on PIR: low income (PIR ≤ 1), middle income (PIR = 1–4), and high income (PIR ≥ 4) (31).Diabetes was determined by self-reported physician diagnosis, use of insulin or oral hypoglycaemic agents, and fasting plasma glucose ≥7.0 mmol/L or HbA1c ≥ 6.5% (32). Cardiovascular disease (including congestive heart failure, coronary heart disease, angina, myocardial infarction and stroke). Smoking status is divided into three categories: never smoker (less than 100 cigarettes), former smoker (100 cigarettes, former smoker) and current smoker (100 cigarettes, current smoker) (33). Drinking status is defined as having consumed alcohol in the past year. Variable information from the NHANES database (https://www.cdc.gov/nchs/nhanes/).

### 2.5 Statistical analysis

To comply with NCHS guidelines, sample weights were applied to statistical estimates to accurately represent the deinstitutionalised US civilian population. Participants’ demographic and baseline characteristics were summarised using the standard deviation mean of continuous variables ± percentage of categorical variables. Means and proportions were compared to assess differences between subjects with and without hypertension.Missing values for variables are listed in Supplementary Table S1.

Continuous variables use multiple interpolation to process missing values, and categorical variables use mode filling to process missing values. There was no significant difference between the data before and after interpolation, indicating that the interpolation program did not change the distribution of variables.

The association was first examined for one variable. The association between ZJU index and hypertension was further analysed by multivariate logistic regression. Three models were used: Model 1 unadjusted; Model 2 adjusted for age, sex and race; Model 3 adjusted for all variables including age, sex, race, educational level, marital status, PIR, smoking status, alcohol consumption, total cholesterol, LDL, HDL, diabetes, cancer, cardiovascular disease.

Restricted cubic spline (RCS) is used to explore potential nonlinear associations between ZJU index and hypertension because of their flexibility and interpretability in modeling dose-response relationships in observational studies.RCS outperforms other nonlinear methods such as polynomials or smoothed spline to avoid overfitting and allow intuitive visualization. Inflection points in nonlinear relationships are identified by recursive algorithms that maximize model likelihood. Specifically, segmented regression models were fitted with candidate nodes and the node that yielded the highest log likelihood was selected as the inflection point. To verify the robustness of the association between ZJU and hypertension, categorical covariates were subgroup analyzed using hierarchical multiple regression, which was combined with an interaction test to explore the variability between different subgroups. The predictive power of the ZJU index to identify hypertension was assessed using receiver operating characteristic (ROC) analysis and area under the curve (AUC) values. A *P* value of 0.05 or less was considered statistically significant. R software (version 4.2.2) (http://www.Rproject.org) and EmpowerStats software (version 6.0) (http://www.empowerstats.com) should be used for all analyses.

## 3 Results

### 3.1 Baseline characteristics of participants

Our analysis included 14,084 participants, of whom 5,976 were hypertensive. Baseline characteristics Table 1 shows significant differences between participants with and without hypertension. Hypertensive patients were older (57.05 vs. 41.77 years, *P* < 0.0001), had higher body mass index (30.97 kg/m^2^ vs. 27.65 kg/m^2^, *P* < 0.0001), fasting glucose, triglycerides, and ZJU index, and lower high-density lipoprotein levels (1.37 mmol/L vs. 1.43 mmol/L, *P* < 0.0001). Hypertension was associated with a higher prevalence of diabetes (24.76% vs. 6.30%), cardiovascular disease (48.59% vs. 19.22%) and cancer (14.52% vs. 6.48%, all *p* < 0.0001). Sociodemographic differences such as educational attainment, marital status and ethnicity were also significant, reflecting the multifactorial etiology of hypertension.

**Table 1:** Baseline characteristics of participants, weighted.

Variable	Hypertension		*P*-value
	No (*n* = 8,108)	Yes (*n* = 5,976)	
Age(years)	41.77 ± 0.53	57.05 ± 0.52	<0.0001
BMI(kg/m^2^)	27.65 ± 0.20	30.97 ± 0.25	<0.0001
TC(mmol/L)	5.02 ± 0.03	5.09 ± 0.04	0.0046
LDL(mmol/L)	2.98 ± 0.03	2.96 ± 0.03	0.2687
HDL(mmol/L)	1.43 ± 0.02	1.37 ± 0.02	<0.0001
FPG(mmol/L)	5.55 ± 0.04	6.33 ± 0.07	<0.0001
TG(mmol/L)	1.37 ± 0.04	1.69 ± 0.05	<0.0001
ALT(U/L)	25.24 ± 0.48	26.52 ± 0.57	0.0013
AST(U/L)	24.90 ± 0.39	26.67 ± 0.55	<0.0001
ZJU index	38.57 ± 0.23	42.96 ± 0.31	<0.0001
Age (%)			<0.0001
<60 years	86.38	53.90	
≥60 years	13.62	46.10	
Gender (%)			0.1247
Male	48.59	49.98	
Female	51.41	50.02	
Race (%)			<0.0001
Mexican American	9.57	5.10	
Other Hispanic	5.53	3.29	
Non-Hispanic White	69.60	73.55	
Non-Hispanic Black	8.57	12.33	
Other Race	6.73	5.73	
Education Level (%)			<0.0001
Below high school	15.09	18.70	
High school	20.95	26.27	
Above high school	63.96	55.03	
Marital status (%)			<0.0001
Married/living with partner	64.68	65.64	
Widowed/divorced/separated	14.08	24.77	
Never married	21.24	9.59	
PIR (%)			0.0145
<1	13.88	12.91	
≥1, <4	48.95	52.38	
≥4	37.17	34.71	
Drinking status (%)			<0.0001
Yes	78.69	72.83	
No	21.31	27.17	
Smoking status (%)			<0.0001
Never	56.01	48.37	
Former	21.72	32.70	
Current	22.28	18.93	
Diabetes (%)			<0.0001
Yes	6.30	24.76	
No	93.70	75.24	
Cardiovascular disease (%)			<0.0001
Yes	19.22	48.59	
No	80.78	51.41	
Cancer (%)			<0.0001
Yes	6.48	14.52	
No	93.52	85.48	

Mean ± SD for continuous variables: the *P* value was calculated by the weighted linear regression model.

(%) for categorical variables: the *P* value was calculated by the weighted chi-square test.

PIR, poverty-income ratio; BMI, body mass index; TC, total cholesterol; LDL, low-density lipoprotein cholesterol; HDL, high-density lipoprotein cholesterol; FPG, fasting blood glucose; TG, triglyceride; ALT, alanine aminotransferase; AST, aspartate aminotransferase.

### 3.2 Association between ZJU index and prevalence of hypertension

The results of our multivariate logistic regression analysis are detailed in Table 2. The ZJU index was significantly and positively associated with the prevalence of hypertension. In model 1, unadjusted for covariates, the OR was 1.07 (95% CI: 1.06–1.07, *p* < 0.0001) per unit increase in ZJU index. In model 3, adjusted for demographic, socioeconomic and clinical factors, the correlation remained significant (OR: 1.07, 95% CI: 1.06–1.07, *p* < 0.0001). When analyzed by quartile, participants in the highest quartile (Q4) had a significantly higher risk of developing hypertension than those in the lowest quartile (Q1) (OR: 3.73, 95% CI: 3.25–4.29, *p* < 0.0001).

**Table 2:** Associations between ZJU index and the prevalence of hypertension.

Exposure	Model 1		Model 2		Model 3	
	OR (95%CI)	*P*-value	OR (95%CI)	*P*-value	OR (95%CI)	*P*-value
ZJU	1.07 (1.06, 1.07)	<0.0001	1.08 (1.07, 1.09)	<0.0001	1.07 (1.06, 1.07)	<0.0001
ZJU categorical						
Q1	1		1		1	
Q2	1.79 (1.61, 1.98)	<0.0001	1.60 (1.42, 1.81)	<0.0001	1.56 (1.38, 1.76)	<0.0001
Q3	2.41 (2.18, 2.67)	<0.0001	2.36 (2.10, 2.66)	<0.0001	2.12 (1.87, 2.41)	<0.0001
Q4	3.97 (3.59, 4.39)	<0.0001	4.89 (4.34, 5.52)	<0.0001	3.73 (3.25, 4.29)	<0.0001
P for trend	<0.0001		<0.0001		<0.0001	

Data are presented as OR, 95% CI and *P*-value.

Model 1 adjust for: none.

Model 2 adjust for: gender, age, race.

Model 3 adjust for: gender; age; race; education level; marital status; PIR; drinking status; smoking status; TC; LDL; HDL; diabetes; CVD; cancer.

**Figure 2:**
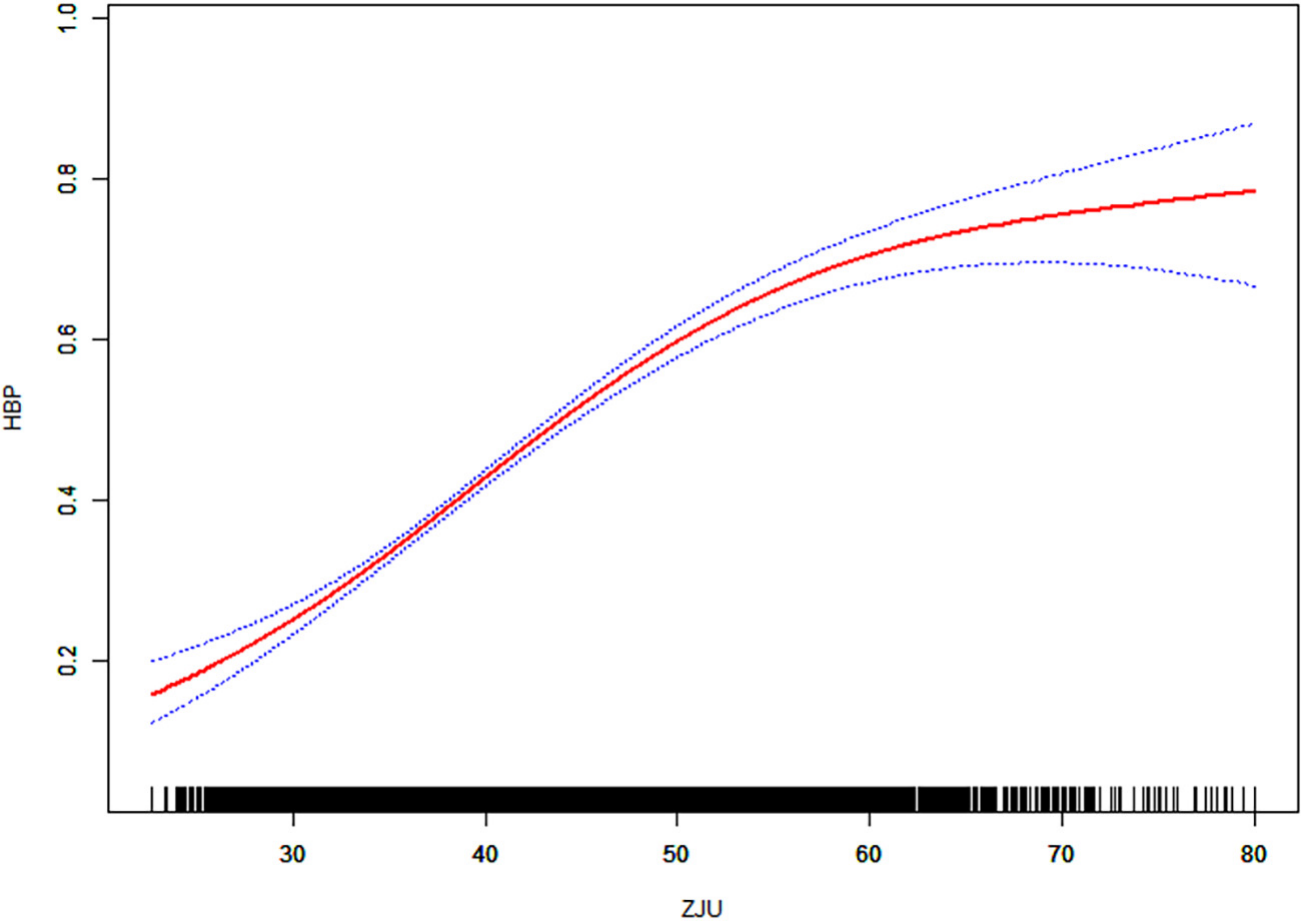
Association between ZJU and hypertension. Adjusted for gender; age; race; education level; marital status; PIR; drinking status; smoking status; TC; LDL; HDL; diabetes; CVD; cancer.

Restricted cubic spline (RCS) was used to characterize the nonlinear relationship between ZJU index and prevalence of hypertension. Threshold effect analyses were fitted using a segmented regression model with candidate nodes, and the node that yielded the highest log likelihood was selected as the inflection point, which was 53.22, as shown in Table 3. Figure 2. The results showed that the risk of hypertension increased nonlinearly with increasing ZJU index, with a clear inflection point at a ZJU value of approximately 53.22. Below this value, the risk increases significantly and the curve steepens, while above 53.22, the risk increases relatively slowly.

**Table 3:** Generalized additive modeling and smoothed curve fitting.

Hypertension	Adjusted OR (95% CI)	*P*-value
ZJU index		
Fitting by the standard linear model	1.07 (1.06, 1.07)	<0.0001
Fitting by the two-piecewise linear model		
Inflection point	53.22	
ZJU index <53.22	1.08 (1.07, 1.09)	<0.0001
ZJU index >53.22	1.02 (1.00, 1.04)	0.0606
P for Log-likelihood ratio	<0.001	

Adjusted as Model 3: gender; age; race; education level; marital status; PIR; drinking status; smoking status; TC; LDL; HDL; diabetes; CVD; cancer.

The area between the upper and lower dashed lines is indicated as the 95% CI. Each point shows the magnitude of the ZJU Index and is connected to form a continuous line. Adjust for all variables (Model 3).

### 3.3 Subgroup analysis

We assessed the stability and heterogeneity of the association between ZJU index and hypertension by subgroup analysis and interaction test analysis. For subgroup analysis, we included all categorical covariates in the study: stratified by gender, age, race, marital status, education, PIR, smoking status, drinking status, diabetes, cancer, and cardiovascular disease. The results are shown in Table 4, where the association between ZJU index and hypertension remained significantly positive in all subgroups. In addition, interaction tests showed significant associations between age, education, smoking status, and diabetes subgroups (interaction *P* < 0.05).

**Table 4:** Subgroup analysis.

Variable	OR (95%CI)	*P* value	P for interaction
Gender			0.1647
Male	1.08 (1.07, 1.09)	<0.0001	
Female	1.07 (1.06, 1.08)	<0.0001	
Age			0.0013
<60	1.07 (1.06, 1.08)	<0.0001	
≥60	1.05 (1.04, 1.06)	<0.0001	
Race			0.3522
Mexican American	1.07 (1.05, 1.09)	<0.0001	
Other Hispanic	1.06 (1.04, 1.08)	<0.0001	
Non-Hispanic White	1.07 (1.06, 1.08)	<0.0001	
Non-Hispanic Black	1.06 (1.05, 1.08)	<0.0001	
Other Race	1.09 (1.06, 1.11)	<0.0001	
Education level			0.0013
Below high school	1.05 (1.04, 1.07)	<0.0001	
High school	1.06 (1.04, 1.08)	<0.0001	
Above high school	1.08 (1.07, 1.09)	<0.0001	
Marital status			0.836
Married/living with partner	1.07 (1.06, 1.08)	<0.0001	
Widowed/divorced/separated	1.07 (1.06, 1.09)	<0.0001	
Never married	1.07 (1.05, 1.08)	<0.0001	
PIR			0.1308
<1	1.06 (1.05, 1.07)	<0.0001	
≥1, <4	1.07 (1.06, 1.08)	<0.0001	
≥4	1.08 (1.07, 1.10)	<0.0001	
Drinking status			0.5894
Yes	1.07 (1.06, 1.08)	<0.0001	
No	1.08 (1.06, 1.09)	<0.0001	
Smoking status			<0.0001
Never	1.09 (1.08, 1.10)	<0.0001	
Former	1.07 (1.05, 1.09)	<0.0001	
Current	1.04 (1.03, 1.06)	<0.0001	
Diabetes			0.0404
Yes	1.06 (1.04, 1.07)	<0.0001	
No	1.08 (1.07, 1.09)	<0.0001	
Cancer			0.4991
Yes	1.06 (1.03, 1.09)	<0.0001	
No	1.07 (1.06, 1.08)	<0.0001	
Cardiovascular disease			0.0828
Yes	1.06 (1.05, 1.08)	<0.0001	
No	1.08 (1.07, 1.09)	<0.0001	

A multivariate logistic regression model was used to adjust all covariates, except for the specific variables used to define each subgroup.

### 3.4 Diagnostic value of ZJU index and its components in hypertension

**Figure 3:**
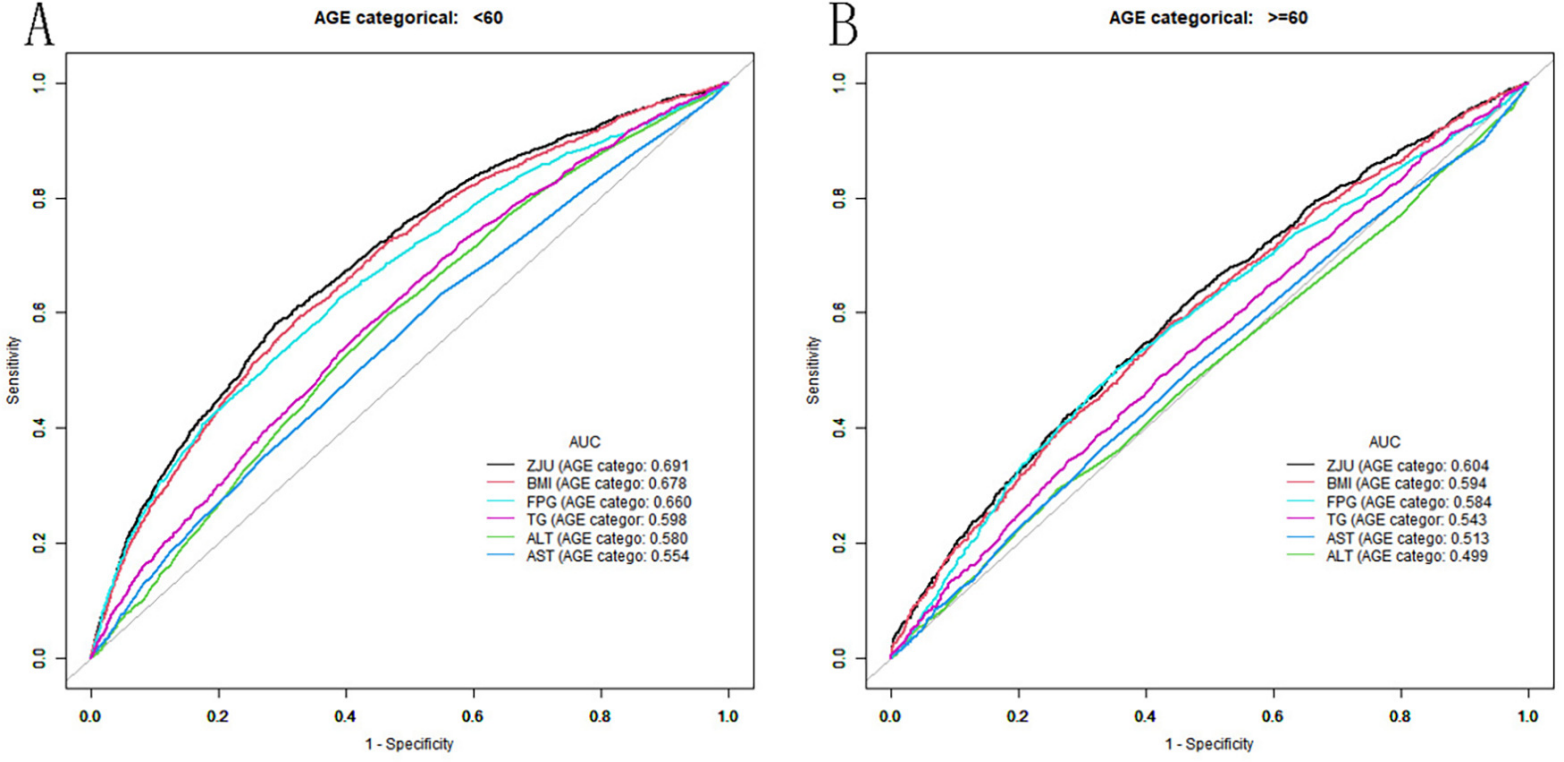
ROC of ZJU index in patients with hypertension (**A**: <60 years old; B: ≥60 years old).

The effectiveness of the ZJU index and its components in discriminating individuals with hypertension was assessed using the ROC curve and AUC values (Figure 3). In a subgroup of participants younger than 60 years, the AUC values of the ZJU index, BMI, FPG, TG, ALT and AST were 0.6911 (0.6792, 0.7031), 0.6782 (0.6661, 0.6903), 0.6602 (0.6476, 0.6729), 0.5982 (0.5854, 0.6109), 0.5797 (0.5669, 0.5924) and 0.5537 (0.5405, 0.5668). It is worth noting that the ZJU index has the highest AUC (Table 5). In contrast, in participants aged 60 years and older, the diagnostic utility of these measures decreased, but the AUC value of the ZJU index remained the highest (AUC = 0.604) (Table 6). In summary, the AUC for the ZJU Index was 0.604 in those over 60 years of age, indicating limited ability to distinguish between hypertensives and non-hypertensives in this subgroup. Although the ZJU Index performed slightly better in participants under 60 years of age (AUC = 0.691), this again represents only a moderate ability to differentiate.

**Table 5:** ROC curve of ZJU index and other related indicators to distinguish hypertension (age <60 years).

Variable	AUC(95%CI)	Best threshold	Specificity	Sensitivity
ZJU	0.6911 (0.6792,0.7031)	41.6659	0.7102	0.5813
BMI	0.6782 (0.6661,0.6903)	29.4750	0.6773	0.5890
FPG	0.6602 (0.6476,0.6729)	5.4595	0.6122	0.6233
TG	0.5982 (0.5854,0.6109)	1.2250	0.5746	0.5701
ALT	0.5797 (0.5669,0.5924)	21.5000	0.5352	0.5975
AST	0.5537 (0.5405,0.5668)	21.5000	0.4517	0.6325

**Table 6:** ROC curve of ZJU index and other related indicators to distinguish hypertension (age ≥60 years).

Variable	AUC(95%CI)	Best threshold	Specificity	Sensitivity
ZJU	0.6044 (0.5870, 0.6217)	38.1272	0.5213	0.6340
BMI	0.5938 (0.5764, 0.6113)	27.5950	0.5619	0.5808
FPG	0.5844 (0.5670, 0.6018)	5.9535	0.6472	0.5003
TG	0.5433 (0.5254, 0.5611)	1.3380	0.5775	0.4905
AST	0.5128 (0.4951, 0.5305)	25.5000	0.6707	0.3633
ALT	0.4991 (0.4815, 0.5167)	24.5000	0.7383	0.2926

Abbreviations: BMI, body mass index; FPG, fasting blood glucose; TG, triglyceride; ALT, alanine aminotransferase; AST, aspartate aminotransferase.

## 4 Discussion

In this study, we analyzed for the first time the association between the ZJU index and hypertension in adults aged 20 years and older in the United States, and showed that the ZJU index was significantly and nonlinearly positively associated with the prevalenceof hypertension. This association was consistently significant in both unadjusted and multivariable-adjusted models. When it was below the threshold of 53.22, for every one-unit increase in the ZJU index, the prevalence of hypertension increased by 8% (OR = 1.08, 95% CI: 1.07–1.09, *P* < 0.0001); When it was above the threshold of 53.22, the prevalence of hypertension increased by 2%, but there was no statistical significance (*P* = 0.0606). Subgroup analyses verified that this association was consistently significant across most demographic and clinical subgroups, and a significant interaction between age, education level, smoking status, and diabetes was observed (*P* < 0.05). Receiver operating characteristic curve (ROC) analysis indicated that the ZJU index had certain potential for predicting the risk of hypertension in the young population (AUC = 0.691), while the correlation weakened in the elderly population (AUC = 0.604), suggesting the need for further research on age-related metabolic changes and their impact on the ZJU index.

The ZJU index integrates several metabolic components, and each reflects a distinct biological pathway potentially implicated in hypertension. Among them, triglycerides (TG) serve as classic markers of disturbed lipid metabolism and have been widely shown to be associated with hypertension risk through mechanisms related to endothelial dysfunction and systemic inflammation (34, 35). Elevated TG not only disrupts nitric oxide bioavailability but also stimulates the release of pro-inflammatory cytokines such as IL-6 and TNF-α, which together compromise vascular health and promote the development of arterial stiffness and higher blood pressure (36–40).

Furthermore, fasting blood glucose (FBG), another component of the ZJU index, indicates glucose metabolism abnormalities and is a surrogate for underlying insulin resistance. Elevated FBG and insulin resistance together can activate both the sympathetic nervous system and the renin-angiotensin-aldosterone system (RAAS), resulting in increased sodium retention, higher peripheral resistance, and ultimately disturbed cardiovascular homeostasis (41–44).Notably, insulin resistance also intensifies oxidative stress, further impairing endothelial function and exacerbating vascular rigidity, which together contribute to the pathophysiological groundwork of hypertension (45, 46).

In addition, BMI, as a measure of overall and central adiposity, represents another important determinant of cardiometabolic risk (47–49). Central obesity leads to excessive secretion of pro-inflammatory mediators like leptin, TNF-α, and IL-6, thus escalating systemic inflammation and vascular injury (50–52). Notably, increased visceral fat further activates the RAAS and enhances aldosterone production, augmenting sodium reabsorption and blood volume, which in turn elevate blood pressure (53, 54). These interrelated pathways underscore the multifactorial influence of obesity on hypertension.

A particularly noteworthy aspect of the ZJU index is the inclusion of the serum ALT/AST ratio, which has garnered attention as a sensitive indicator of liver metabolic status and a potential link to broader cardiometabolic risk. Recent studies have highlighted that an elevated ALT/AST ratio, commonly suggestive of non-alcoholic fatty liver disease (NAFLD), is associated not only with hepatic dysfunction, but also with systemic metabolic disturbances, including insulin resistance and chronic low-grade inflammation (55–58). Importantly, NAFLD and elevated liver enzymes can trigger oxidative stress and the release of pro-inflammatory cytokines, thereby exacerbating endothelial dysfunction and arterial stiffness. Hepatic metabolic stress may thus contribute to an unfavorable vascular milieu, reducing the capacity for blood pressure regulation and promoting the onset of hypertension (53, 59, 60). Moreover, accumulating evidence suggests the ALT/AST ratio is closely linked to dysregulated lipid and glucose metabolism—central features underlying hypertension pathogenesis (61, 62). By reflecting this liver-metabolic axis, the ALT/AST ratio serves as more than a liver marker: it is a bridge connecting hepatic, metabolic, and vascular health within the context of hypertension risk.

Moreover, sex differences are systematically accounted for in the ZJU index to reflect known epidemiological and physiological disparities in hypertension prevalence. Premenopausal women, for instance, commonly possess a lower risk of hypertension compared to men, primarily attributable to estrogen's protective effects on vascular endothelium and regulation of oxidative stress and RAAS activity (63–65). However, with menopause, the reduction of estrogen and subsequent rise in visceral adiposity reverses this advantage, heightening women's susceptibility to hypertension. Concurrently, men are often at higher risk of hypertension at younger ages, a trend linked to greater visceral fat accumulation and increased oxidative stress following puberty (66, 67). Through sex adjustment, the ZJU index can adaptively accommodate these distinct physiological characteristics across diverse groups.

Despite the ZJU index's comprehensive construction, its predictive capability for hypertension remains moderate in our analysis. In particular, the index possessed better discriminative value in younger adults (AUC = 0.691) than in older individuals (AUC = 0.604), and only a modest improvement over simpler measures such as BMI (AUC = 0.678) was observed. Consequently, the added complexity introduced by using the integrated index must be weighed against its incremental benefits in practice. Furthermore, our results showed that when compared to established risk stratification tools, such as the Framingham hypertension risk score, the ZJU index did not demonstrate superior predictive performance in our study population (68, 69). Therefore, these findings indicate the necessity for further refinement and external validation of the ZJU index before it can be widely applied in clinical settings.

Strengths of this study include the use of a large, nationally representative sample, providing strong evidence for the potential applicability of the ZJU index in different populations. In addition, the analysis of nonlinear associations and identification of key thresholds provided useful insights for public health and clinical applications. The consistency of results across most subgroups highlights the versatility of the index in capturing metabolic risk factors in populations. However, several limitations must be recognized. First, as a cross-sectional study, reverse causality cannot be completely ruled out; that is, hypertension itself may affect metabolic indices, rather than inversely, and longitudinal studies are needed to confirm the temporal dynamics of this association. Second, despite adjustment for multiple covariates, residual confounders of unmeasured factors (e.g., unrecorded lifestyle variables or genetic susceptibility) may still be present and influence the observed associations. Third, the reduced diagnostic relevance of the ZJU index in older adults suggests that it may not fully capture age-specific hypertension risk and that further refinements or additional markers are needed for this population. Despite these limitations, this study provides a valuable basis for further research and highlights the potential utility of the ZJU index in hypertension risk stratification and management.

## 5 Conclusion

In this nationally representative cross-sectional study, we observed a significant association between the ZJU index and hypertension risk in adults, with stronger associations present among younger individuals. However, as this analysis is observational in nature, no causal relationships can be established. Future research should focus on prospective cohort studies to assess the temporal and predictive value of the ZJU index in hypertension development, as well as intervention trials to evaluate whether risk stratification or management based on the ZJU index improves outcomes. Additionally, studies on index optimization for different populations may enhance its clinical utility.

## Data Availability

The original contributions presented in the study are included in the article/Supplementary Material, further inquiries can be directed to the corresponding author.

## References

[1] Sayer M Webb DJ Dhaun N. Novel pharmacological approaches to lowering blood pressure and managing hypertension. Nat Rev Cardiol. (2025). 10.1038/s41569-025-01131-4 39920248

[2] He S Park S Kuklina E Therrien NL Lundeen EA Wall HK Leveraging electronic health records to construct a phenotype for hypertension surveillance in the United States. Am J Hypertens. (2023) 36:677–85. 10.1093/ajh/hpad081 37696605 PMC10898654

[3] Fan Z Yang C Zhang J Huang Y Yang Y Zeng P Trends and influence factors in the prevalence, awareness, treatment, and control of hypertension among US adults from 1999 to 2018. PLoS One. (2023) 18:e0292159. 10.1371/journal.pone.0292159 37768964 PMC10538741

[4] Wang C Yuan Y Zheng M Pan A Wang M Zhao M Association of age of onset of hypertension with cardiovascular diseases and mortality. J Am Coll Cardiol. (2020) 75:2921–30. 10.1016/j.jacc.2020.04.038 32527401

[5] Jadhav UM Ray S Unni TG Sawhney JPS Mehta A Guha S Expert opinion on the role of sacubitril/valsartan in the management of hypertension in India. Cardiol Ther. (2024) 13:663–77. 10.1007/s40119-024-00390-5 39503972 PMC11607345

[6] Yaacoub S Boudaka A AlKhatib A Pintus G Sahebkar A Kobeissy F The pharmaco-epigenetics of hypertension: a focus on microRNA. Mol Cell Biochem. (2024) 479:3255–71. 10.1007/s11010-024-04947-9 38424404 PMC11511726

[7] Cho JS Park J-H. Application of artificial intelligence in hypertension. Clin Hypertens. (2024) 30(1):11. 10.1186/s40885-024-00266-9 38689376 PMC11061896

[8] Gnjidic D Langford AV Jordan V Sawan M Sheppard JP Thompson W Withdrawal of antihypertensive drugs in older people. Cochrane Database Syst Rev. (2025) 3:CD012572. 10.1002/14651858.CD012572.pub3 40162571 PMC11956142

[9] Felkle D Jarczyński M Kaleta K Zięba K Nazimek K. The immunomodulatory effects of antihypertensive therapy: a review. Biomed Pharmacother. (2022) 153:113287. 10.1016/j.biopha.2022.113287 35728352

[10] Dzau VJ Hodgkinson CP. Precision hypertension. Hypertension. (2024) 81:702–8. 10.1161/HYPERTENSIONAHA.123.21710 38112080

[11] Grave C Bonaldi C Carcaillon-Bentata L Gabet A Halimi J-M Tzourio C Burden of cardio-cerebrovascular and renal diseases attributable to systolic hypertension in France in 2021. Hypertension. (2025) 82:357–69. 10.1161/HYPERTENSIONAHA.124.23760 39648886

[12] Kaur S Rubal Kaur S Kaur A Kaur S Gupta S A cross-sectional study to correlate antioxidant enzymes, oxidative stress and inflammation with prevalence of hypertension. Life Sci. (2023) 313:121134. 10.1016/j.lfs.2022.121134 36544300

[13] Li G Xu S Messerlian C Zhang Y Chen Y-J Sun Y Blood trihalomethane and urinary haloacetic acid concentrations in relation to hypertension: an observational study among 1162 healthy men. J Hazard Mater. (2024) 477:135411. 10.1016/j.jhazmat.2024.135411 39111173

[14] Xu J-P Zeng R-X Zhang Y-Z Lin S-S Tan J-W Zhu H-Y Systemic inflammation markers and the prevalence of hypertension: a NHANES cross-sectional study. Hypertens Res. (2023) 46:1009–19. 10.1038/s41440-023-01195-0 36707716

[15] Chen J Wang B Liu C Li C Meng T Wang J Association between platelet to high-density lipoprotein cholesterol ratio (PHR) and hypertension: evidence from NHANES 2005–2018. Lipids Health Dis. (2024) 23:346. 10.1186/s12944-024-02342-3 39462374 PMC11514891

[16] Son D-H Lee HS Lee Y-J Lee J-H Han J-H. Comparison of triglyceride-glucose index and HOMA-IR for predicting prevalence and incidence of metabolic syndrome. Nutr Metab Cardiovasc Dis. (2022) 32:596–604. 10.1016/j.numecd.2021.11.017 35090800

[17] Adams-Huet B Zubirán R Remaley AT Jialal I. The triglyceride-glucose index is superior to homeostasis model assessment of insulin resistance in predicting metabolic syndrome in an adult population in the United States. J Clin Lipidol. (2024) 18(4):e518–24. 10.1016/j.jacl.2024.04.130 38834412

[18] Zeng X Han D Zhou H Xue Y Wang X Zhan Q Triglyceride-glucose index and homeostasis model assessment-insulin resistance in young adulthood and risk of incident congestive heart failure in midlife: the coronary artery risk development in young adults study. Front Cardiovasc Med. (2022) 9:944258. 10.3389/fcvm.2022.944258 35845059 PMC9279654

[19] Bulut M Celik FB Guvenc TS Yilmaz Y Celik M Ozyildirim S Usefulness of triglyceride-glucose index and homeostatic model assessment for predicting coronary microvascular dysfunction. J Clin Lipidol. (2024) 18:e764–72. 10.1016/j.jacl.2024.04.135 38955587

[20] Zheng K Yin Y Guo H Ma L Liu R Zhao T Association between the ZJU index and risk of new-onset non-alcoholic fatty liver disease in non-obese participants: a Chinese longitudinal prospective cohort study. Front Endocrinol (Lausanne). (2024) 15:1340644. 10.3389/fendo.2024.1340644 38405152 PMC10884868

[21] Wang J Xu C Xun Y Lu Z Shi J Yu C ZJU index: a novel model for predicting nonalcoholic fatty liver disease in a Chinese population. Sci Rep. (2015) 5:16494. 10.1038/srep16494 26568423 PMC4645098

[22] Liao Y-Y Wang D Chu C Man Z-Y Wang Y Ma Q Long-term burden and increasing trends of body mass index are linked with adult hypertension through triglyceride-glucose index: a 30-year prospective cohort study. Nutr Metab Cardiovasc Dis. (2024) 34:2134–42. 10.1016/j.numecd.2024.05.014 39003135

[23] Tsai K-Z Chu C-C Huang W-C Sui X Lavie CJ Lin G-M. Prediction of various insulin resistance indices for the risk of hypertension among military young adults: the CHIEF cohort study, 2014–2020. Cardiovasc Diabetol. (2024) 23:141. 10.1186/s12933-024-02229-8 38664804 PMC11046748

[24] Zhu L Fang Z Jin Y Chang W Huang M He L Association between serum alanine and aspartate aminotransferase and blood pressure: a cross-sectional study of Chinese freshmen. BMC Cardiovasc Disord. (2021) 21:472. 10.1186/s12872-021-02282-1 34598675 PMC8485510

[25] Liu H Zha X Ding C Hu L Li M Yu Y AST/ALT ratio and peripheral artery disease in a Chinese hypertensive population: a cross-sectional study. Angiology. (2021) 72:916–22. 10.1177/00033197211004410 33779311

[26] Liu H Ding C Hu L Li M Zhou W Wang T The association between AST/ALT ratio and all-cause and cardiovascular mortality in patients with hypertension. Medicine (Baltimore). (2021) 100:e26693. 10.1097/MD.0000000000026693 34397804 PMC8341222

[27] Ma X Zou H Zhan J Gao J Xie Y. Assessment of the clinical value of five noninvasive predictors of metabolic dysfunction-associated steatotic liver disease in Han Chinese adults. Eur J Gastroenterol Hepatol. (2024) 36:1209–19. 10.1097/MEG.0000000000002806 38973526

[28] Wu C Loh YH Huang H Xu C. ZJU index as a predictive tool for diabetes incidence: insights from a population-based cohort study. Diabetes Metab Syndr Obes. (2024) 17:715–24. 10.2147/DMSO.S446042 38371391 PMC10873143

[29] Hao J-Q Hu S-Y Zhuang Z-X Zhang J-W Xiong M-R Wang R The ZJU index is associated with the risk of sarcopenia in American adults aged 20–59: a cross-sectional study. Lipids Health Dis. (2024) 23:389. 10.1186/s12944-024-02373-w 39593075 PMC11590360

[30] Zhang X Wei R Wang X Zhang W Li M Ni T The neutrophil-to-lymphocyte ratio is associated with all-cause and cardiovascular mortality among individuals with hypertension. Cardiovasc Diabetol. (2024) 23:117. 10.1186/s12933-024-02191-5 38566082 PMC10985955

[31] Zhao Y Zhao J Xie R Zhang Y Xu Y Mao J Association between family income to poverty ratio and HPV infection status among U.S. women aged 20 years and older: a study from NHANES 2003–2016. Front Oncol. (2023) 13:1265356. 10.3389/fonc.2023.1265356 37860196 PMC10582625

[32] Dong G Gan M Xu S Xie Y Zhou M Wu L. The neutrophil-lymphocyte ratio as a risk factor for all-cause and cardiovascular mortality among individuals with diabetes: evidence from the NHANES 2003–2016. Cardiovasc Diabetol. (2023) 22:267. 10.1186/s12933-023-01998-y 37775767 PMC10541705

[33] Li Y Xia P-F Geng T-T Tu Z-Z Zhang Y-B Yu H-C Trends in self-reported adherence to healthy lifestyle behaviors among US adults, 1999 to march 2020. JAMA Netw Open. (2023) 6:e2323584. 10.1001/jamanetworkopen.2023.23584 37450300 PMC10349344

[34] Yang S Zhang Y Zhou Z Duan X. Association of triglyceride-glucose index, triglyceride to high-density lipoprotein cholesterol ratio, and related parameters with prehypertension and hypertension. J Clin Hypertens (Greenwich). (2025) 27:e14926. 10.1111/jch.14926 39447019 PMC11774080

[35] Lin Y-H Liu Y-H Wu D-W Su H-M Chen S-C. Dyslipidemia increases the risk of incident hypertension in a large Taiwanese population follow-up study. Nutrients. (2022) 14:3277. 10.3390/nu14163277 36014784 PMC9416084

[36] Yang P-T Li Y Wang J-G Zhang L-J Yang S-Q Tang L The association of remnant cholesterol with endothelial dysfunction and subclinical atherosclerosis in a check-up population in China. J Atheroscler Thromb. (2023) 30:684–97. 10.5551/jat.63695 36104205 PMC10244076

[37] Mendiola PJ Morin EE Gonzalez Bosc LV Naik JS Kanagy NL. Role of cholesterol in the regulation of hydrogen sulfide signaling within the vascular endothelium. Antioxidants (Basel). (2022) 11:1680. 10.3390/antiox11091680 36139754 PMC9495961

[38] Ren Z Guo J Xiao X Huang J Li M Cai R The effect of sex differences on endothelial function and circulating endothelial progenitor cells in hypertriglyceridemia. Cardiol Res Pract. (2020) 2020:2132918. 10.1155/2020/2132918 33014455 PMC7526329

[39] Yu L Xu L Chu H Peng J Sacharidou A Hsieh H-H Macrophage-to-endothelial cell crosstalk by the cholesterol metabolite 27HC promotes atherosclerosis in male mice. Nat Commun. (2023) 14:4101. 10.1038/s41467-023-39586-z 37491347 PMC10368733

[40] Guo D-C Gao J-W Wang X Chen Z-T Gao Q-Y Chen Y-X Remnant cholesterol and risk of incident hypertension: a population-based prospective cohort study. Hypertens Res. (2024) 47:1157–66. 10.1038/s41440-023-01558-7 38212367

[41] Hamaoka T Leuenberger UA Drew RC Murray M Blaha C Luck JC Glucose metabolism and autonomic function in healthy individuals and patients with type 2 diabetes mellitus at rest and during exercise. Exp Physiol. (2024) 109:214–26. 10.1113/EP091444 38050866 PMC10841625

[42] Smith EC Patel JN Wahba A Cluckey A Celedonio J Park J Acute sympathetic blockade improves insulin-mediated microvascular blood flow in the forearm of adult human subjects with obesity. J Am Heart Assoc. (2024) 13:e030775. 10.1161/JAHA.123.030775 39119951 PMC11963928

[43] Del Bianco V Ferreira G Bochi APG Pinto PR Rodrigues LG Furukawa LNS Aerobic exercise training protects against insulin resistance, despite low-sodium diet-induced increased inflammation and visceral adiposity. Int J Mol Sci. (2024) 25:10179. 10.3390/ijms251810179 39337664 PMC11432465

[44] Monea G Jiritano R Salerno L Rubino M Massimino M Perticone M Compromised cardiac autonomic function in non-diabetic subjects with 1h post-load hyperglycemia: a cross-sectional study. Cardiovasc Diabetol. (2024) 23:295. 10.1186/s12933-024-02394-w 39127733 PMC11316982

[45] Jia G Sowers JR. Hypertension in diabetes: an update of basic mechanisms and clinical disease. Hypertension. (2021) 78:1197–205. 10.1161/HYPERTENSIONAHA.121.17981 34601960 PMC8516748

[46] Hill MA Yang Y Zhang L Sun Z Jia G Parrish AR Insulin resistance, cardiovascular stiffening and cardiovascular disease. Metab Clin Exp. (2021) 119:154766. 10.1016/j.metabol.2021.154766 33766485

[47] Fu L Cheng H Zhao X Hou D Xie X Mi J. Distinct causal effects of body fat distribution on cardiometabolic traits among children: findings from the BCAMS study. Nutr Metab Cardiovasc Dis. (2022) 32:1753–65. 10.1016/j.numecd.2022.03.030 35599089

[48] Georgoulis M Damigou E Chrysohoou C Barkas F Kravvariti E Tsioufis C Increased body weight and central adiposity markers are positively associated with the 20-year incidence of cardiovascular disease: the ATTICA epidemiological study (2002–2022). Nutr Res. (2024) 121:1–15. 10.1016/j.nutres.2023.10.008 37995411

[49] Haregu TN Nanayakkara S Carrington M Kaye D. Prevalence and correlates of normal body mass index central obesity among people with cardiovascular diseases in Australia. Public Health. (2020) 183:126–31. 10.1016/j.puhe.2020.03.013 32497780

[50] Cobos-Palacios L Muñoz-Úbeda M Gallardo-Escribano C Ruiz-Moreno MI Vilches-Pérez A Vargas-Candela A Adipokines profile and inflammation biomarkers in prepubertal population with obesity and healthy metabolic state. Children (Basel). (2022) 9:42. 10.3390/children9010042 35053667 PMC8774044

[51] Popko K Gorska E Stelmaszczyk-Emmel A Plywaczewski R Stoklosa A Gorecka D Proinflammatory cytokines il-6 and TNF-α and the development of inflammation in obese subjects. Eur J Med Res. (2010) 15(Suppl 2):120–2. 10.1186/2047-783x-15-s2-120 21147638 PMC4360270

[52] Flores Gomez D Bekkering S Ter Horst R Cossins B van den Munckhof ICL Rutten JHW The effect of leptin on trained innate immunity and on systemic inflammation in subjects with obesity. J Leukoc Biol. (2024) 115:374–84. 10.1093/jleuko/qiad118 37776323

[53] Packer M. Leptin-Aldosterone-Neprilysin axis: identification of its distinctive role in the pathogenesis of the three phenotypes of heart failure in people with obesity. Circulation. (2018) 137:1614–31. 10.1161/CIRCULATIONAHA.117.032474 29632154

[54] Quadri SS Culver S Ramkumar N Kohan DE Siragy HM. (Pro)Renin receptor mediates obesity-induced antinatriuresis and elevated blood pressure via upregulation of the renal epithelial sodium channel. PLoS One. (2018) 13:e0202419. 10.1371/journal.pone.0202419 30118514 PMC6097690

[55] Zou Y Zhong L Hu C Sheng G. Association between the alanine aminotransferase/aspartate aminotransferase ratio and new-onset non-alcoholic fatty liver disease in a nonobese Chinese population: a population-based longitudinal study. Lipids Health Dis. (2020) 19:245. 10.1186/s12944-020-01419-z 33239040 PMC7690093

[56] Liu H Li H Deng G Zheng X Huang Y Chen J Association of AST/ALT ratio with 90-day outcomes in patients with acute exacerbation of chronic liver disease: a prospective multicenter cohort study in China. Front Med (Lausanne). (2024) 11:1307901. 10.3389/fmed.2024.1307901 38576715 PMC10993385

[57] Niu A Qi T. Diagnostic significance of serum type IV collagen (IVC) combined with aspartate aminotransferase (AST)/alanine aminotransferase (ALT) ratio in liver fibrosis. Ann Transl Med. (2022) 10:1310. 10.21037/atm-22-5010 36660657 PMC9843355

[58] Xuan Y Wu D Zhang Q Yu Z Yu J Zhou D. Elevated ALT/AST ratio as a marker for NAFLD risk and severity: insights from a cross-sectional analysis in the United States. Front Endocrinol (Lausanne). (2024) 15:1457598. 10.3389/fendo.2024.1457598 39253584 PMC11381241

[59] Méndez-García LA Escobedo G Baltazar-Pérez I Ocampo-Aguilera NA Arreola-Miranda JA Cid-Soto MA Exploring the Th2 response in obesity and metabolic dysfunction-associated steatotic liver disease (MASLD): a potential modulator of the renin-angiotensin system (RAS) pathway in hypertension development. Life (Basel). (2024) 14:1080. 10.3390/life14091080 39337863 PMC11433558

[60] Yuan M He J Hu X Yao L Chen P Wang Z Hypertension and NAFLD risk: insights from the NHANES 2017–2018 and Mendelian randomization analyses. Chin Med J (Engl). (2024) 137:457–64. 10.1097/CM9.0000000000002753 37455323 PMC10876227

[61] Visaria A Pai S Cheung M Ahlawat S. Association between aspartate aminotransferase-to-alanine aminotransferase ratio and insulin resistance among US adults. Eur J Gastroenterol Hepatol. (2022) 34:316–23. 10.1097/MEG.0000000000002215 34074988

[62] Higashitani M Mizuno A Kimura T Shimbo T Yamamoto K Tokioka S Low aspartate aminotransferase (AST)/alanine aminotransferase (ALT) ratio associated with increased cardiovascular disease and its risk factors in healthy Japanese population. J Gastrointestin Liver Dis. (2022) 31:429–36. 10.15403/jgld-4446 36535061

[63] Rethy L Polsinelli VB Muntner P Bello NA Cohen JB. Association of blood pressure variability with endothelin-1 by menopause status among black women: findings from the Jackson heart study. J Hum Hypertens. (2023) 37:742–5. 10.1038/s41371-023-00824-y 36966225 PMC11097102

[64] Heravi AS Michos ED Zhao D Ambale-Venkatesh B De Vasconcellos H D Lloyd-Jones D Oxidative stress and menopausal Status: the coronary artery risk development in young adults cohort study. J Womens Health (Larchmt). (2022) 31:1057–65. 10.1089/jwh.2021.0248 35675673 PMC9299529

[65] Stamellou E Sterzer V Alam J Roumeliotis S Liakopoulos V Dounousi E. Sex-Specific differences in kidney function and blood pressure regulation. Int J Mol Sci. (2024) 25:8637. 10.3390/ijms25168637 39201324 PMC11354550

[66] Chao H Hu Y Wang Q Tang B Adji A Avolio A Impact of obesity phenotype on central aortic hemodynamics and arterial stiffness in a Chinese health assessment population. Rev Cardiovasc Med. (2022) 23:216. 10.31083/j.rcm2306216 39077167 PMC11273641

[67] García-Sánchez A Gómez-Hermosillo L Casillas-Moreno J Pacheco-Moisés F Campos-Bayardo TI Román-Rojas D Prevalence of hypertension and obesity: profile of mitochondrial function and markers of inflammation and oxidative stress. Antioxidants (Basel). (2023) 12:165. 10.3390/antiox12010165 36671026 PMC9854635

[68] Jung TY Kim MS Hong HP Kang KA Jun DW. Comparative assessment and external validation of hepatic steatosis formulae in a community-based setting. J Clin Med. (2020) 9:2851. 10.3390/jcm9092851 32899243 PMC7565459

[69] Koohi F Steyerberg EW Cheraghi L Abdshah A Azizi F Khalili D. Validation of the framingham hypertension risk score in a middle eastern population: Tehran lipid and glucose study (TLGS). BMC Public Health. (2021) 21:790. 10.1186/s12889-021-10760-6 33894756 PMC8070324

