# Supplementary material for: Nonlinear association of a composite metabolic index (ZJU index) with hypertension: a cross-sectional study of NHANES 2003–2018
Source: Front Cardiovasc Med. 2025 Jun 18;12:1608648. doi: 10.3389/fcvm.2025.1608648 (PMC12213919; doi:10.3389/fcvm.2025.1608648)
Supplement: Supplementary file 1 [file Table1.docx]

**Table S1.** The number and percentage of missing values.

| Variables | n(%) |
| --- | --- |
| Marital status | 3(0.02) |
| Education Level | 9(0.06) |
| Smoking Status | 10(0.07) |
| LDL-cholesterol | 281(1.55) |
| Diabetes | 215(1.53) |
| Cardiovascular disease | 1(0.007) |
| Cancer | 11(0.78) |
